# The effect of family integrated care on preparing parents with premature infants hospitalized in the neonatal intensive care unit for discharge

**DOI:** 10.1038/s41372-024-01931-5

**Published:** 2024-03-18

**Authors:** Öznur Tiryaki, Nursan Çınar, İbrahim Caner

**Affiliations:** 1 Department of Midwifery, Faculty of Health Science, Sakarya University, Sakarya, Turkey; 2 Department of Pediatric Nursing, Faculty of Health Science, Sakarya University, Sakarya, Turkey; 3 Faculty of Medicine, Division of Neonatology, Department of Pediatrics, Sakarya University, Sakarya, Turkey

**Keywords:** Paediatrics, Patient education

## Abstract

**Objective:**

The study was designed as a randomized controlled experimental trial to determine the effect of the Family Integrated Care (FICare) model on the readiness of parents whose infants were hospitalized in the neonatal intensive care unit (NICU) for discharge and home care of the infants.

**Study design:**

Parents in the intervention group received FICare, and parents in the control group received standard care.

**Results:**

The total mean score of the mothers and fathers in the intervention group regarding readiness for discharge and home care was higher than that of the control group, and a significant difference was observed. A statistically significant difference was found in terms of discharge weight, the day of first enteral feeding, and first breast milk.

**Conclusion:**

The FICare model was observed to enhance the readiness of mothers and fathers for discharge and home care and positively affect the infant’s weight gain, the status of breastfeeding and the continuation of nutrition.

**Clinical trial registration:**

Registered on ClinicalTrials.gov (Identifiers: NCT04478162 Unique Protocol ID: 16214662/050.01.04/14) on 17/07/2020.

## Introduction

The Family Integrated Care (FICare) model was designed to eliminate the barriers between parents and infants by involving parents, whose premature infants were hospitalized in the neonatal intensive care unit (NICU), in the care of their infants [1, 2]. FICare encourages parents to assume primary caregiving roles in infants’ non-medical usual care, in which healthcare professionals in the NICU and parents have common responsibilities [3]. The four basic components of the FICare model are the education of parents, the education of the NICU personnel, physical arrangements in the NICU, and providing psychosocial support to parents [4–6]. The FICare model is a modern approach that supports the participation of parents in infant care (excluding ventilation, monitor adjustment, intravenous fluid, and medication administration) provided stage by stage in the NICU and is developed through the collaboration between parents and healthcare professionals [7]. Parents are informed about infants’ general development, brain and sensory development, motor and behavioral development, and care of premature infants (especially touching, attachment, skin-to-skin contact, breastfeeding, changing diapers, etc.) [8]. The FICare model emerged in low-income societies (Estonia) with the idea of trying to meet the need for nurses by including parents in care due to the increasing workload resulting from the insufficient number of nurses [9]. Parents were enabled to take roles in the care of their infants through the educational and mentoring support of nurses. The FICare model, which has received significant interest in many countries, was evaluated in a randomized controlled trial in North America, Australia, and New Zealand, and it was emphasized that nurses played a key role in the successful implementation of the model [10, 11]. It is extremely important to increase the knowledge level of nurses and to strengthen nursing care to provide better quality care to premature babies and their parents in the NICU [12, 13]. Parents of premature infants will feel closer to their infants when they receive education and counseling support from well-trained neonatal nurses, and a positive relationship will develop between parents and nurses. Hence, parents’ satisfaction with the NICU will increase, and they will be able to trust themselves more while performing their parenting roles [14]. It is known that the FICare model increases the baby’s weight gain and breastfeeding, and reduces the mother’s stress and anxiety [1, 7]. When the literature was examined, no study was found regarding parents’ readiness for discharge in NICUs where the FICare model was applied.

### Aim

This study was conducted to investigate the effect of FICare, which was applied for the first time in Turkey, on the level of readiness of mothers and fathers whose premature infants were hospitalized in the NICU for discharge and home care.

### Hypothesis

H_0_: There is no difference between the readiness for discharge of mothers and fathers included in the FICare model compared to the control group.

H_1_: Discharge readiness of mothers included in the FICare model is higher than the control group.

H_2_: Discharge readiness of fathers included in the FICare model is higher than the control group.

## Method

### Study design

This was a randomized controlled trial that was registered in the Registry of Clinical Trials (code: NCT04478162). Premature infants and their parents were included in the control and intervention groups. The FICare model was applied to the parents of premature infants in the intervention group, and the NICU standard care was applied to the parents of premature infants in the control group.

### Sample

Premature infants who were hospitalized for at least 7 days between February 6, 2020, and August 15, 2021, in the NICU of a training and research hospital formed the population of the study. The NICU of the hospital has a capacity of 42 beds (2nd and 3rd level), and ~700 newborns are hospitalized annually. The participants of the study were calculated in accordance with the intervention group selection criteria and by performing power analysis. Power analysis was performed using GPower (v3.1.7) program. The effect range value was taken as 0.70 as the method used in cases where it is unknown how many units difference is significant between the groups. In cases where Type 1 error probability (a) was 0.05 (at a confidence level of 95%), at a power level of 80% and the effect range was 0.70, the study was planned with a total of 68 parents, including 34 parents for each group. At the end of the study, a post hoc power analysis was conducted to determine the adequacy of the sample size. As a result of the power analysis, for the details of the difference between the groups in terms of the scale score of fathers with premature babies in the neonatal intensive care unit, type 1 error: 0.05, *n*: 68 people, effect size = 1.990, and the power level according to the structure was determined as 1.000. To determine the difference in terms of the scale score of mothers with premature babies in the neonatal intensive care unit, type 1 error: 0.05, *n*: 68 people, effect size = 2.586 and power level according to temperature was determined as 1.000. These values show that the sample size is sufficient [15].

**Fig. 1:**
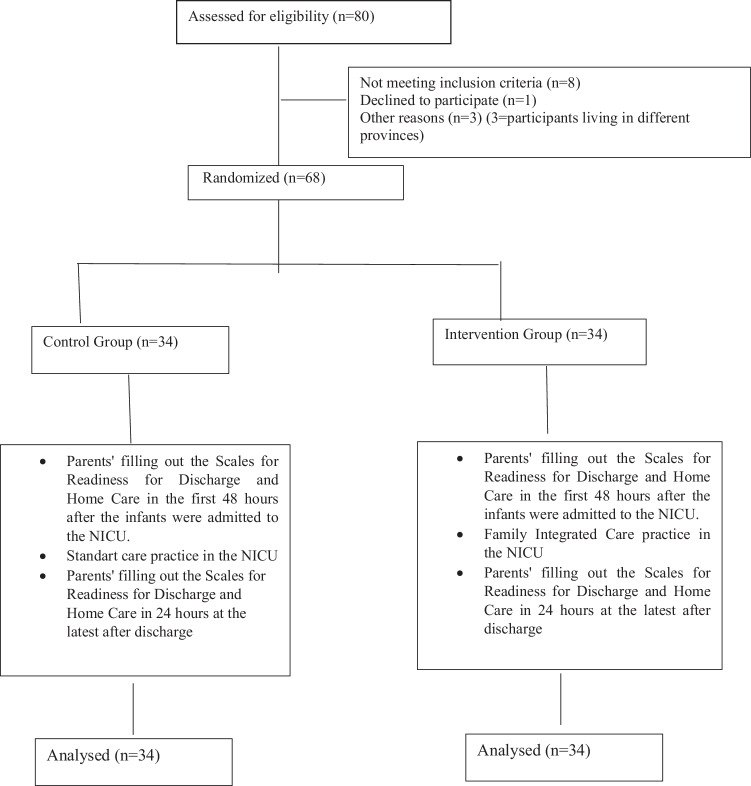
CONSORT flow diagram of the study.

Of individuals meeting the inclusion criteria, 34 were assigned to the intervention group and 34 to the control group (Fig. 1). Premature infants and their parents were included in the control and intervention groups. Blinding could not be done to prevent the groups using the same mother’s hotel from being affected by each other. Therefore, the data of first the control group and then the intervention group were collected. “Control” and “Intervention” were written in closed envelopes to determine which group to start with first. The nurse in charge of the NICU, who was not the author of the study, was asked to choose an envelope. Since the standard care group appeared in the first selected envelope, it started to work with this group first. The FICare model was applied to the parents of premature infants in the intervention group, and the NICU standard care was applied to the parents of premature infants in the control group.

The inclusion criteria for preterm infants were as follows:

1. Birth of the infant at the 28th–34th weeks of gestation,
2. First parenting experience of the mother and the father,
3. Parents’ willingness to participate in the study and being open to communication,
4. Mother having breast milk,
5. Staying in the NICU for at least 1 week,
6. Participation of parents in the discharge training program.

The exclusion criteria for preterm infants were as follows:

1. Undergoing a surgical intervention,
2. Death of the baby,
3. Transfer of the baby to another hospital,
4. Failure of the parent to complete all phases of the study.

### Procedure

The forms used in the study were filled out within the first 48 h after the birth when the mothers and fathers in the groups felt fine and could establish healthy communication with the researcher (XX). The study was terminated by having the same forms filled out again within 24 h at the latest before discharge.

### Intervention

All phases of the FICare model have been implemented. Parents in the intervention group were included in a 1-week training program within the scope of the Family Integrated Care model. A maximum of four couples attended the training in each session. A training program was also organized at weekend for those who could not attend it during the week. Training subjects consisted of the importance of breast milk, breastfeeding positions, hygienic care practices (eyes, nose, mouth, ears, skin, diaper change), bathing, nail clipping, kangaroo care, drug administration, first and emergency support, safe sleep, doctor check-up times, and vaccine follow-ups. Care practices were first shown on the model infant, and parents were asked to practice on the model. If parents did not understand certain points, they would be allowed to meet one-to-one every Monday morning. An average of 3-h training on FICare was given to healthcare personnel in the NICU. Moreover, a 4-h training on Family Integrated Care practice was organized for nurses working in the NICU. The process was coordinated by a senior nurse in the NICU. Parents who completed their training were enabled, with the support of nurses in the NICU, to take care of their infants and practice until they could do it on their own. When the clinical stabilization of their infants was achieved, parents were asked to attend at least three caregiving sessions and stay in the hospital for an average of 6–8 h. While preparations for discharge continued, peer interviews were held with experienced parents whose premature infants had been discharged from the NICU before. At discharge, a guide was given as a summary of the training program to facilitate the home transition. Furthermore, manual milking was taught in the first 6 h after birth, and the milking and breastfeeding process was supported by a lactation counselor.

### Control

Individuals received standard care provided by nurses from the time the premature infant was admitted to the NICU until discharge. The usual care process is carried on between the nurse and the mother. Mothers are allowed to perform limited care practices (bottom cleaning, breastfeeding) that the nurse considers appropriate. Mothers of babies who are planned to be discharged start staying in the hospital ~2 days before. Fathers are only informed and not included in the care.

### Measures and instrumentations

The discharge weight of the infants in the groups, the number of days spent in the NICU, the first breast milk intake, breastfeeding for the first time, and the time of transition to full enteral feeding were evaluated. The total scores of the scales applied to parents were calculated.

### Instruments

#### Mother, father, and infant descriptive information form

The form includes questions about the mother’s/father’s age, education level, employment status, income status, family type, mode of delivery, infant’s sex, birth week, birth weight, discharge weight, feeding process in the NICU, and the number of days spent in the NICU [1, 2, 7, 8].

#### The scale for the readiness of the mother with a premature infant in the neonatal intensive care unit for discharge and home care

Developed by Tiryaki and Çınar [16], the scale consists of 22 positive items of seven-point Likert type and four (feeding, perception of general condition, hygienic care, care practices) sub-scales. Each item in the scale is scored between 1 and 7 from, and the total scale score is obtained with the sum of the scores. A minimum score of 22 and a maximum score of 154 are obtained from the scale, and high scores indicate that parents’ readiness for discharge is high. The total variance of the scale is 72.886%. In the study in which the scale was developed, the intraclass correlation coefficient for mothers was found to be 0.835. The Cronbach’s alpha value of the developed scale was found to be 0.911.

#### The scale for the readiness of the father with a premature infant in the neonatal intensive care unit for discharge and home care

Developed by Tiryaki and Çınar [16], the scale consists of 20 positive items of seven-point Likert type and three (feeding and care support, hygienic care, care practices) sub-scales. Each item in the scale is scored between 1 and 7 from, and the total scale score is obtained with the sum of the scores. A minimum score of 20 and a maximum score of 144 are obtained from the scale, and high scores indicate that parents’ readiness for discharge is high. The total variance of the scale is 67.36%. In the study in which the scale was developed, the intraclass correlation coefficient for fathers was found to be 0.942. The Cronbach’s alpha value of the developed scale was found to be 0.948.

#### Ethical issues

Permission was obtained from the institution where the study would be conducted and from the Clinical Research Ethics Committee of Sakarya University Faculty of Medicine (Approval number: 16214662/050.01.04/14). Verbal and written consent was received from the parents of premature infants after informing them about the purpose of the study, the design of the study, and how the data would be used.

### Data analysis

The data were transferred to the IBM SPSS Statistics 23 program and completed. While evaluating the study data, frequency distribution (number, percentage) was given for categorical variables and descriptive statistics (mean, standard deviation) for numerical variables. The independent sample *t*-test was used to check whether there was a difference between the groups. The chi-square test was conducted to examine the relationship between the groups and categorical variables. Moreover, the dependent sample *t*-test was applied to examine changes over time. *p* < 0.05 was considered significant.

## Results

The mean age of the mothers in the intervention group was 27.97 ± 4.86 years. The mean age of the mothers in the control group was 27.21 ± 5.64 years. The mean age of the fathers in the intervention group was 31.35 ± 4.43 years. The mean age of the fathers in the control group was 31.26 ± 5.26 years. The gender of 58.8% of the infants in the intervention group was female, and the mean gestational week was 31.50 ± 2.12. The gender of 44.1% of the infants in the control group was female, and the mean gestational week was 31.53 ± 1.62. There was no statistically significant difference between the groups in terms of demographic characteristics of mothers, fathers, and infants (*p* > 0.05) (Table 1).

**Table 1:** Examination of the relationship between the groups and the demographic characteristics of the mother, father, and infant.

		Intervention			Control			Test	*p
		*N* = 34		%	*N* = 34		%		
Information about the mother									
Age (mean ± sd)		27.97 ± 4.86			27.21 ± 5.64			0.598^t^	0.552
Educational background	Secondary education	25		73.5	27		79.4	0.327^k^	0.567
	Higher education	9		26.5	7		20.6		
Employment status	Yes	17		50.0	17		50.0	0.000^k^	1.000
	No	17		50.0	17		50.0		
Family type	Nuclear	30		88.2	32		94.1	0.731^k^	0.393
	Extended	4		11.8	2		5.9		
Place of residence	Province	14		41.2	20		58.8	2.118^k^	0.146
	District/Village	20		58.8	14		41.2		
Age of marriage (mean ± sd)		25.47 ± 4.65			24.91 ± 5.31			0.462^t^	0.646
Total number of pregnancies	1	24		70.6	22		64.7	0.269^k^	0.604
	2 and above	10		29.4	12		35.3		
How pregnancy occurred	Spontaneously	28		82.4	32		94.1	2.267^k^	0.132
	Through IVF or insemination	6		17.6	2		5.9		
Mode of delivery	Normal birth	9		26.5	10		29.4	0.073^k^	0.787
	Cesarean section	25		73.5	24		70.6		
Information about the father									
Age (mean ± sd)		31.35 ± 4.43			31.26 ± 5.26			0.075^t^	0.941
Educational background	Secondary education	21	61.8		21	61.8		0.000^k^	1.000
	Higher education	13	38.2		13	38.2			
	District/Village	17	50.0		11	32.4			
Information about the infant									
Infant’s sex	Female	20	58.8		15	44.1		1.472^k^	0.225
	Male	14	41.2		19	55.9			
Week of gestation (mean ± sd)		31.50 ± 2.12			31.53 ± 1.62			−0.064^t^	0.949

*k* chi-square test, *t* independent sample *t*-test, *sd* standard deviation.

*p < 0.05.

According to calculations, infants in the intervention group started enteral feeding on day 1.88 ± 1.12, taking breast milk on day 2.15 ± 1.97, and sucking breast milk on day 12.76 ± 9.37 for the first time. Full enteral feeding was started on day 16.59 ± 11.65, the average number of days spent in the NICU was 27.59 ± 19, and the average discharge weight was 2137.79 ± 370.10 grams. Infants in the control group started enteral feeding on day 3.38 ± 1.72, taking breast milk on day 4.18 ± 1.75, and sucking breast milk on day 18.47 ± 13.61 for the first time. Full enteral feeding was started on day 20.26 ± 14.93, the average number of days spent in the NICU was 30.18 ± 20.54, and the average discharge weight was 1965.59 ± 285.50 grams. While there was no statistically significant difference between the groups in terms of the number of days spent in the NICU and full enteral feeding start day *t* = −0.539, *p* = 0.592; *t* = −1.132, *p* = 0.262), a statistically significant difference was observed in terms of discharge weight, enteral feeding starts day, first breast milk intake, and breastfeeding for the first time (*t* = 2.148, *p* = 0.035; *t* = −4.253, *p* < 0.0001; *t* = −4.490, *p* < 0.0001; *t* = −2.013, *p* = 0.048) (Table 2).

**Table 2:** Comparison of the intervention and control groups in terms of some variables (*N* = 68).

	Intervention		Control		*t*	*p*
	Mean	sd	Mean	sd		
Discharge weight	2137.79	370.10	1965.59	285.50	2.148	0.035*
Number of days spent in the NICU	27.59	19.00	30.18	20.54	−0.539	0.592
Number of MV days^a^	1.94	2.82	2.79	3.87	−1.039	0.302
Number of CPAP days^b^	4.24	4.02	3.56	2.46	0.837	0.406
Enteral feeding start day	1.88	1.12	3.38	1.72	−4.253	0.000*
Full enteral feeding start day	16.59	11.65	20.26	14.93	−1.132	0.262
Time of the first breast milk intake	2.15	1.97	4.18	1.75	−4.49	0.000*
Time of breastfeeding for the first time	12.76	9.37	18.47	13.61	−2.013	0.048*

*t* independent sample *t*-test, *sd* standard deviation.

*p < 0.05.

^a^Mechanical ventilator.

^b^Continuous positive airway pressure.

Due to the evaluation of “the scale for the readiness of the mother with a premature infant in the neonatal intensive care unit for discharge and home care” (*t*_1_) filled out by the mothers in the intervention group within the first 48 h after giving birth, when they felt fine, the mean total score received from the scale was 93.56 ± 16.45, whereas the mean total score received from the scale (*t*_1_) by the mothers in the control group was 86.68 ± 24.86. Within the scope of the FICare model, mothers who were getting ready for discharge in the intervention group were made to fill out the scale again within 24 h at the latest before the discharge of their infants (*t*_2_). The mean total score received from the scale by the mothers in the intervention group was 145.26 ± 8.54, and the mean total score of the mothers in the control group was 111.24 ± 16.53 (Table 3).

**Table 3:** Comparison of mothers and fathers with premature infants in the neonatal intensive care unit in terms of the scale and dimension scores of readiness for discharge and home care by group.

	Intervention (*N*:34)		Control (*N*:34)		*t*_*a*_	*p*
	Mean	sd	Mean	sd		
Information about the mother						
Feeding (*t*_1_)	12.68	3.67	12.00	4.09	0.717	0.476
Feeding (*t*_2_)	20.00	1.33	16.50	2.89	6.410	0.000*
*t*_*b*_/*p*	−12.213/0.000*		−7.831/0.000*			
Perception of the general condition (*t*_1_)	16.18	4.18	14.62	5.23	1.358	0.179
Perception of the general condition (*t*_2_)	25.82	2.10	19.24	4.11	8.335	0.000*
*t*_*b*_/*p*	−15.697/0.000*		−7.753/0.000*			
Hygienic care (*t*_1_)	25.71	7.05	24.35	9.56	0.664	0.509
Hygienic care (*t*_2_)	45.24	3.69	30.62	7.62	10.07	0.000*
*t*_*b*_/*p*	−16.700/0.000*		−6.006/0.000*			
Care practices (*t*_1_)	39.00	7.50	35.71	8.50	1.695	0.095
Care practices (*t*_2_)	54.21	3.43	44.88	5.37	8.534	0.000*
*t*_*b*_/*p*	−11.323/0.000*		−6.257/0.000*			
The scale for readiness of the mother with a premature infant in the neonatal intensive care unit for discharge and home care (*t*_1_)	93.56	16.45	86.68	24.86	1.346	0.183
The scale for readiness of the mother with a premature infant in the neonatal intensive care unit for discharge and home care (*t*_2_)	145.26	8.54	111.24	16.53	10.666	0.000*
*t*_*b*_/*p*	−18.013/0.000*		−8.098/0.000*			
Information about the father						
Support for feeding and care (*t*_1_)	28.06	6.86	25.74	9.85	1.129	0.263
Support for feeding and care (*t*_2_)	48.06	3.82	35.06	9.33	7.519	0.000*
*t*_*b*_/*p*	−15.906/0.000*		−7.639/0.000*			
Hygienic care (*t*_1_)	17.47	7.53	17.88	10.02	−0.192	0.849
Hygienic care (*t*_2_)	39.32	4.4	24.62	10.41	7.585	0.000*
*t*_*b*_/*p*	−15.322/0.000*		−5.629/0.000*			
Care practices (*t*_1_)	23.18	6.72	21.09	7.31	1.227	0.224
Care practices (t_2_)	31.12	2.85	26.38	5.40	4.523	0.000*
*t*_*b*_/*p*	−7.459/0.000*		−7.421/0.000*			
The scale for readiness of the father with a premature infant in the neonatal intensive care unit for discharge and home care (*t*_1_)	68.71	16.51	64.71	23.66	0.808	0.422
The scale for readiness of the father with a premature infant in the neonatal intensive care unit for discharge and home care (*t*_2_)	118.50	9.67	86.06	20.93	8.205	0.000*
*t*_*b*_/*p*	−16.678/0.000*		−8.076/0.000*			

*t*_1_ before care practice; *t*_2_ after care practice.

*t*_*a*_ independent sample *t*-test, *t*_*b*_ dependent sample *t*-test, *sd* standard deviation.

*p < 0.05.

The results show that of “the scale for the readiness of the father with a premature infant in the neonatal intensive care unit for discharge and home care” (*t*_1_) filled out by the fathers in the intervention group within the first 48 h after the infants’ admission to the NICU, when they felt fine and at the most appropriate time to establish communication, the mean total score received from the scale was 68.71 ± 16.51, whereas the mean total score received from the scale by the fathers in the control group was 64.71 ± 23.66. Within the scope of the FICare model, fathers who were getting ready for discharge in the intervention group were made to fill out “the scale for the readiness of the father with a premature infant in the neonatal intensive care unit for discharge and home care” again within 24 h at the latest before the discharge of their infants (*t*_2_). The mean total score received from the scale by the fathers in the intervention group was 118.50 ± 9.67, and the mean total score of the fathers in the control group was 86.06 ± 8.205 (Table 3). According to Table 3, there was no statistically significant difference between the groups of mothers and fathers in terms of pre-test measurements (*p* = 0.183, *t* = 1.346; *p* = 0.422, *t* = 0.808), while there was a statistically significant difference in terms of post-test measurements (*t* = 10.666, *p* < 0.0001; *t* = 8.205, *p* < 0.0001).

## Discussion

It is important to evaluate the readiness of parents who are responsible for the primary care of babies after discharge, before they leave the hospital, to ensure the safety of the baby in the home environment and to improve their quality of health care [17]. Readiness for discharge requires parents to be emotionally comfortable and self-confident, and to feel ready for parenthood, in addition to learning technical knowledge and skills about baby care [18]. In our study, which included mothers and fathers with first-time parenting experience whose premature babies were hospitalized in the NICU, the readiness scores of parents who were applied the FICare care model were found to be higher than those of parents who received standard care. Infants’ first enteral feeding start time, first breast milk intake time, and breastfeeding for the first time took place earlier in the intervention group than in the control group. The discharge weights of the infants in the intervention group were higher than those in the control group. There was no significant difference between the groups in terms of the day’s infants spent in the NICU, the number of MV days, the number of CPAP days, and the full enteral feeding start day.

When the two groups of infants with very low birth weights (VLBW) in the NICU who received and did not receive colostrum within the first 12 h were compared, it was observed that the infants who received colostrum had an earlier transition to full enteral feeding, a shorter TPN period. While the results were similar to our study, the average hospitalization day of infants with VLBWs in the NICU in the same study was found to be higher in the intervention group than in the control group, unlike our study [19]. In a study examining the nutrition of premature infants at discharge, 71.9% of the infants were discharged with exclusive breastfeeding. It was emphasized that skin contact between the mother and the infant and the earlier start of milking increased the rates of infants’ sucking and breast milk intake [20].

In a study comparing FICare and standard care, it was stated that the time of parents’ hugging their infants for the first time and the time of transition to full enteral feeding took place earlier in the FICare group [21]. In another study conducted in the UK, it was concluded that premature infants who received FICare were discharged from the NICU earlier, and breastfeeding and full enteral feeding were started earlier [22]. In the randomized controlled trial conducted in 11 NICUs in China, it was found that premature infants in the FICare group stayed in the hospital for a shorter time, resulting in reduced medical expenditures, faster weight gain, lower infection, and antibiotic use rate, and higher rates of breastfeeding and breast milk intake [23]. In the international randomized controlled trial in which FICare was provided in tertiary NICUs, it was revealed that the stress and anxiety of parents decreased, and infants’ development, weight gain, and breastfeeding rates increased [1]. First breast milk intake, breastfeeding, and transition to full enteral feeding occurred earlier, and infants gained better weight in our study. When these findings were compared to studies on the FICare model, similar results were obtained. We think that the use of the model, even in different cultures, has a positive effect on the health of premature infants and their parents.

A peer meeting was organized by bringing together the parents in the intervention group and the parents whose premature infants had been hospitalized and discharged from the NICU. While experienced parents expressed their feelings by explaining the processes they lived through during the meeting, parents in the intervention group were allowed to ask questions. Similar to our study, it was stated that the peer meeting held with the parents of premature infants who had been discharged from the NICU before and infants who were still hospitalized was a good practice to express feelings [24]. Among the parent education topics in FICare, breastfeeding, nutrition, and usual care practices of infants were found to be the most interesting topics for parents [25].

Discharge training for parents with infants in the NICU is the most important component of FICare [26]. Discharge training should be initiated as soon as possible after the infant is admitted to the unit and continued until parents are ready to take their infants home [27]. The evaluation of parents’ readiness is important to ensure that parents can provide the necessary infant care independently and safely [28]. A reliable tool is needed to provide a comprehensive pre-discharge evaluation of parents’ readiness for the discharge of their infants. Thus, parents’ needs can be determined before discharge, and necessary preventive interventions can be planned and implemented accordingly [17]. Considering the nature of discharge training, it was emphasized that the strongest predictor of readiness for discharge was a comprehensive discharge preparation program. Parents should be provided with an individualized and systematic educational approach. This will help especially infants at high risk to have a healthier home care process after discharge from the NICU [27]. In a study, it was emphasized that parents were generally not prepared for the transition from hospital to home care, and situations such as physical fatigue, change in lifestyle, and adaptation to the parenting role were the sources of stress. High-quality discharge training, which is the most important component of the discharge preparation process and given by nurses, will help the home transition process be more moderate and help parents prepare for discharge in premature infant care [29]. The FICare model makes it easy to identify their training needs and provide the support they need [26]. In evaluating the effectiveness of the FICare care model, the program can be evaluated by using scales. The use of practical scales will help us evaluate parents objectively in questioning their readiness for discharge and home care. In line with the low scores received from the scale items, parents can be supported in matters they feel inadequate in.

### Limitations and strengths

One challenge of the study is that part of the data collection phase overlapped with a period of pandemic-related restrictions on NICU visitation. During this period, data collection was suspended, and the NICU started the data collection process again after the restrictions were lifted. This situation prolonged the data collection process. Another difficulty of the research is that the number of training sessions is higher than planned. Since some of the fathers in the study worked during the day, training and practices were arranged according to the fathers’ working hours. Additional sessions were organized on weekdays and weekends to facilitate fathers’ participation. The results of the study are limited to data obtained from participants who had a baby between 28–34 weeks and experienced parenthood for the first time.

The strengths of the study include the use of the FICare care model for the first time in Turkey, the inclusion of the hard-to-reach father sample group in the study, and their active participation in the care of their babies. Additionally, since the researchers have over 10 years of NICU experience, establishing communication with unit staff and parents facilitated the conduct of the study. Another strength is that the members of the support group, which consists of parents who have a premature or sick baby and whose babies have previously been in the NICU, talk about their own experiences and start a conversation around common themes with the parents who attend the session. Information is provided from experienced parents about how they cope with anxiety and difficulties.

## Conclusions

In the study comparing the FICare model and standard care practices of parents with premature infants in the NICU, the mean scores received by mothers and fathers from the scale for readiness for discharge and home care are higher in the FICare group. First breast milk intake, first breastfeeding, and transition to full enteral feeding of the infants in the FICare group was observed earlier than the control group. The weights of the infants whose discharge was planned are also higher in the intervention group.

It is recommended that a trained and competent nurse give discharge training to t parents of infants who are planning to be discharged from the NICU, and even that a discharge training nurse be determined. Having a breastfeeding counselor nurse within the NICU will be effective in starting and sustaining breast milk intake and breastfeeding. For parents of infants who need special care and support, a preparatory class for the parenting newborns at risk can be organized, and training sessions can be held on the needed topics. This is the first study in which the FICare model was applied in Turkey. Monitoring post-discharge follow-ups of infants and parents receiving FICare service in a company with longitudinal studies (time of breast milk intake, neuromotor development of infants, attachment, etc.) will shed light on the literature. The sample of this study consisted of participants who experienced parenthood for the first time. It is recommended to conduct comparative studies with parents who have previous parenting experience or with premature babies under 28 weeks.

## Data Availability

The datasets used and analyzed during the current study are available from the corresponding author on reasonable request.
